# Medication Overuse Headache Successfully Treated by Three Types of Japanese Herbal Kampo Medicine

**DOI:** 10.7759/cureus.16800

**Published:** 2021-07-31

**Authors:** Masahito Katsuki, Shin Kawamura, Kenta Kashiwagi, Akihito Koh

**Affiliations:** 1 Department of Neurosurgery, Itoigawa General Hospital, Itoigawa, JPN; 2 Department of Neurology, Itoigawa General Hospital, Itoigawa, JPN

**Keywords:** goshuyuto (tj-31), goreisan (tj-17), kakkonto (tj-1), kampo medicine (japanese herbal medicine), medication overuse headache (moh), primary headache, alternative therapy

## Abstract

Medication overuse headache (MOH) usually resolves after the overuse is stopped and starting prophylactic medications. However, it can be challenging to prescribe common prophylactic medications when patients have a history of side effects. As an alternative therapy, traditional Japanese herbal kampo medicine can be used. We herein report a case of a MOH woman with a history of side effects by such common prophylactic medications. A 50-year-old woman presented with a severe migraine attack. She had suffered from migraines for 10 years. She had taken loxoprofen and sumatriptan every day for over eight years. As prophylactic medications, lomerizine, valproic acid, and amitriptyline had been prescribed in the past, but they were discontinued due to side effects. Therefore, she could continue only propranolol as prophylactic medication. She had severe pulsatile headaches and nausea every day. We diagnosed triptan- and non-steroidal anti-inflammatory drug-overuse headache (the International Classification of Headache Disorders 3rd edition code 8.2.2 and 8.2.3.2) and chronic migraine (code 1.3). She was admitted and stopped loxoprofen and sumatriptan. We prescribed three types of Japanese herbal kampo medicines - kakkonto (TJ-1), goreisan (TJ-17), and goshuyuto (TJ-31). Her headache was relieved on day 5, and she was discharged on day 7. In the 40 days after discharge, she had only three times mild headaches with a numeric rating scale (NRS) of 2/10. She did not need any triptans nor anti-inflammatory drugs. We herein presented the MOH woman who was successfully treated using three types of kampo medicine. We should pay attention to their side effects, but kampo medicine may be useful for MOH treatment as acute and prophylactic medications for primary headaches.

## Introduction

Medication overuse headache (MOH), the International Classification of Headache Disorders 3rd edition (ICHD-3) code 8.2, is a condition in which headache occurring on 15 or more days per month in a patient with a pre-existing primary headache and developing as a consequence of regular overuse of acute or symptomatic headache medication (on 10 or more or 15 or more intakes days per month, depending on the medication) for more than three months. The annual prevalence of MOH is 1%-2%, and MOH is common in middle-aged and women. The headache usually resolves after the overuse is stopped [1]. According to the Japanese Clinical Practice Guideline for Chronic Headache 2013 [2], the treatment strategy is as follows: (1) discontinue the overused medication, (2) treat the headache after discontinuing the overused medication, and (3) administer prophylactic medications.

However, it can be challenging to prescribe common prophylactic medications like anticonvulsants, antihypertensive drugs, or antidepressants when patients have a history of side effects from those prophylactic medications, such as hypotension, drowsiness, and allergies. As an alternative therapy, traditional Japanese herbal kampo medicine can be used [2,3]. We herein report a MOH woman with a history of side effects from such common prophylactic medications. Her MOH was successfully relieved by kampo medicine instead of the common prophylactic medications.

## Case presentation

A 50-year-old woman presented with a severe migraine attack with a numeric rating scale (NRS) of 10/10 and vomiting. She had suffered from migraines since the age of 40. She had taken three-times intakes of 60-mg loxoprofen and a single intake of 50-mg sumatriptan every day over eight years. For the previous year, she had needed a 3-mg subcutaneous sumatriptan injection once a month. As prophylactic medications, lomerizine, valproic acid, and amitriptyline had been prescribed in the past, but they were discontinued due to side effects, such as dizziness, drowsiness, and rash. Therefore, she could continue only 10-mg propranolol three times a day. She had severe pulsatile headache and nausea every day, and its severity was as NRS 6-10/10.

**Figure 1:**
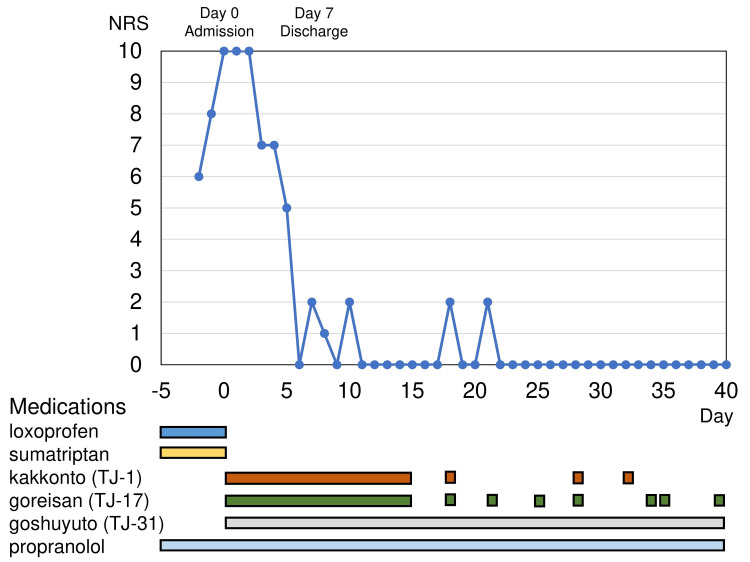
Headache severity and the medication intake After stopping loxoprofen and sumatriptan, the severity gradually improved. In addition to propranolol, kakkonto (TJ-1), goreisan (TJ-17), and goshuyuto (TJ-31) acted as acute and prophylactic medication. NRS; numerical rating scale for pain.

We diagnosed triptan-overuse headache (ICHD-3 code 8.2.2), non-steroidal anti-inflammatory drug-overuse headache (8.2.3.2), and chronic migraine (1.3). She was admitted to our hospital for a severe headache. Her blood pressure was 128/70 mmHg, heart rate was 72 bpm, and body temperature was 36.8℃. The laboratory tests and magnetic resonance imaging did not reveal any particular findings. We stopped loxoprofen and sumatriptan as a treatment for MOH. Considering the history of the side effects using the common prophylactic medications, we prescribed three packets of kakkonto (TJ-1) [4], goreisan (TJ-17) [5,6], and goshuyuto (TJ-31) [7,8] per day. These kampo medicines are sometimes used in Japanese clinical practice and described as an alternative therapy in the Japanese Clinical Practice Guideline for Chronic Headache 2013 [2]. She also started keeping the headache diary. Her headache was relieved on day 5, and she was discharged on day 7. She continued the three types of kampo medicines and propranolol for seven days after discharge, and then she continued propranolol and three packets of goshuyuto (TJ-31) a day and took a pack of kakkonto (TJ-1) and goreisan (TJ-17) as needed. In the 40 days after discharge, she had only three times mild headaches with NRS 2/10, which could be relieved by kakkonto (TJ-1) and goreisan (TJ-17) intake as needed. They are also used as prophylactic medications, and she took them when she felt a headache coming on. She did not need any triptans nor anti-inflammatory drugs in these 40 days, and we will adequately decrease these kampo medicines over the next six months (Figure 1).

## Discussion

We herein presented the MOH woman who was successfully treated using three types of kampo medicine. We herein review the previous report on kampo medicine for MOH and discuss how kampo medicines act as acute and prophylactic medications for headaches.

Kampo medicine for MOH

Akaishi [9] reported a 42-year-old woman with MOH using ibuprofen, migraine without aura (ICHD-3 code 1.1), and chronic tension-type headache (2.3). Triptans and valproic acid were ineffective. Within a month from starting tokishakuyakusan (TJ-23), her headache was gradually improved, and she could stop ibuprofen. Tokishakuyakusan is a traditional herbal medicine frequently prescribed to women with dysmenorrhea or menopausal disorder. Tokishakuyakusan is hypothesized to function by increasing the secretion of estrogen and progesterone from the ovary, possibly by activating the hypothalamic‐pituitary‐gonadal axis. The authors suggested that tokishakuyakusan could be a useful alternative treatment for premenopausal women suffering from severe headaches.

Mitsufuji [10] reported a 20-year-old woman with MOH using ibuprofen and migraine. The doctors suggested prophylactic medications, but the patient refused. She seemed to have anxiety and depression, so they prescribed yokukansan (TJ-54). Within two months, she could stop ibuprofen, and the anxiety and depression also disappeared. Most of the common prophylactic medications cannot be used for women who wish to be pregnant, so yokukansan is relatively safe for such young women. Mitsufuji also reported two other MOH cases that were not successfully treated using common prophylactic medications. The patients were treated using yokukansan in addition to anticonvulsants and anxiolytics [11]. The mechanism of how yokukansan acts for MOH is unknown. Mitsufuji hypothesized that yokukansan suppresses the microglia’s activation in the spinal tract in the trigeminal nerve, which is important for the development of chronic migraine from episodic migraine [10].

These are some of the few reports on MOH patients treated by kampo medicine. Our case, treated by three types of kampo medicine, may suggest another MOH treatment strategy using kampo medicine.

Kakkonto (TJ-1) for headache

Kakkonto (TJ-1) is made up of seven herbal ingredients. It is effective for headache, shoulder stiffness, and muscle pain, presumably because its herbal ingredients perform each effect; mao has adrenergic effects that improve capillary circulation in the muscles, shakuyaku has an analgesic effect, kanzo has an anti-inflammatory effect, and keihi warms up the body. In addition, some of these effects are basically investigated and demonstrated in animal and in vitro experimental models [12]. These effects may have favorable effects on headaches, particularly tension-type headaches by upregulating blood flow in the shoulder muscles. Further basic research is needed.

Goreisan (TJ-17) for headache

Goreisan (TJ-17) is composed of five herbal components and is used to adjust the water balance of the body by inhibiting mainly aquaporin 4 (AQP4) [13] channels as well as other AQP subtypes. AQP3, 4, and 5 upregulate chemokine production [14], and goreisan has a potential anti-inflammatory effect by inhibiting AQPs [15]. Also, there is a glymphatic system in the cranium, which drains inflammatory substances, suppresses inflammation and pain, protects the brain, and prevents brain edema. The gatekeeper of glymphatic flow is AQP4, which exists in the endfeet of astrocytes and serves as a channel to ameliorate the glymphatic system by adjusting water balance in tissues and blood vessels. When cortical spreading depression occurs in migraine, inflammatory substances are released, causing a headache. Then, glymphatic flow is suppressed, which worsens and continues the headache. Goreisan may activate the glymphatic flow correctly and promote the drainage of inflammatory substances, thereby suppressing the headache [16,17].

Goshuyuto (TJ-31) for headache

Goshuyuto (TJ-31) is composed of four herbal components. In basic research, goshuyuto inhibits platelet aggregation in guinea-pig whole blood [18] and constricts the isolated rat aorta [19]. These findings suggested that goshuyuto decreases platelet hyper-aggregation, preventing excess serotonin release. It also constricts vessels appropriately, avoiding acute relaxation of the blood vessel constriction. These are important for migraine attacks. In clinical research, after 12 weeks of treatment by goshuyuto for its responder with chronic headache, the blood serotonin level increased [8]. These findings suggest that goshuyuto adjusts serotonin levels and vessel constriction appropriately and that raising serotonin levels beforehand by goshuyuto may suppress the hyper-reactivity of serotonin receptors for serotonin, leading to the prevention of migraine attacks.

Side effects of kampo medicines

Kampo medicines have been used empirically for headaches and have shown therapeutic effects [2,20], but they also have side effects. The adverse effects of immune-allergic reactions include interstitial pneumonia, liver injury, allergic cystitis, and drug eruption. In our three types of kampo medicine, kakkonto (TJ-1) contains kanzo and mao. Pseudoaldosteronism caused by kanzo and sympathomimetic symptoms caused by mao should be careful as side effects [21].

## Conclusions

We herein presented the MOH woman who was successfully treated using three types of kampo medicine. Kakkonto (TJ-1) may have favorable effects on headaches, possibly by upregulating blood flow in the shoulder muscles. Goreisan (TJ-17) has a potential anti-inflammatory effect by inhibiting AQPs and adjusting glymphatic flow to improve intracranial inflammation. Goshuyuto (TJ-31) prevents the excess serotonin release, and it constricts vessels appropriately, avoiding acute relaxation of the blood vessel constriction. We should pay attention to their side effects, but kampo medicine may be useful for MOH treatment as acute and prophylactic medications for primary headaches.
